# Radiation treatment monitoring using multimodal functional imaging: PET/CT (^18^F-Fluoromisonidazole & ^18^F-Fluorocholine) and DCE-US

**DOI:** 10.1186/s12967-015-0708-5

**Published:** 2015-12-18

**Authors:** Natalia Arteaga-Marrero, Cecilie Brekke Rygh, Jose F. Mainou-Gomez, Tom C. H. Adamsen, Nataliya Lutay, Rolf K. Reed, Dag R. Olsen

**Affiliations:** Department of Physics and Technology, University of Bergen, P.O. Box 7803, Bergen, 5020 Norway; Department of Biomedicine, University of Bergen, Bergen, Norway; Department of Health Sciences, Bergen University College, Bergen, Norway; Department of Clinical Medicine, University of Bergen, Bergen, Norway; Department of Radiology, Haukeland University Hospital, Bergen, Norway; Department of Chemistry, University of Bergen, Bergen, Norway; Division of Dermatology and Venereology, Department of Clinical Sciences, Lund University, Lund, Sweden; Centre for Cancer Biomarkers (CCBIO), University of Bergen, Bergen, Norway

**Keywords:** PET/CT, ^18^F-Fluoromisonidazole, ^18^F-FMISO, ^18^F-Fluorocholine, ^18^F-FCH, DCE-US, CWR22

## Abstract

**Background:**

This study aims to assess the effect of radiation treatment on the tumour vasculature and its downstream effects on hypoxia and choline metabolism using a multimodal approach in the murine prostate tumour model CWR22. Functional parameters derived from Positron Emission Tomography (PET)/Computer Tomography (CT) with ^18^F-Fluoromisonidazole (^18^F-FMISO) and ^18^F-Fluorocholine (^18^F-FCH) as well as Dynamic Contrast-Enhanced Ultrasound (DCE-US) were employed to determine the relationship between metabolic parameters and microvascular parameters that reflect the tumour microenvironment. Immunohistochemical analysis was employed for validation.

**Methods:**

PET/CT and DCE-US were acquired pre- and post-treatment, at day 0 and day 3, respectively. At day 1, radiation treatment was delivered as a single fraction of 10 Gy. Two experimental groups were tested for treatment response with ^18^F-FMISO and ^18^F-FCH.

**Results:**

The maximum Standardized Uptake Values (SUVmax) and the mean SUV (SUVmean) for the ^18^F-FMISO group were decreased after treatment, and the SUVmean of the tumour-to-muscle ratio was correlated to microvessel density (MVD) at day 3. The kurtosis of the amplitude of the contrast uptake *A* was significantly decreased for the control tumours in the ^18^F-FCH group. Furthermore, the eliminating rate constant of the contrast agent from the plasma *k*_*el*_ derived from DCE-US was negatively correlated to the SUVmean of tumour-to-muscle ratio, necrosis and MVD.

**Conclusions:**

The present study suggests that the multimodal approach using ^18^F-FMISO PET/CT and DCE-US seems reliable in the assessment of both microvasculature and necrosis as validated by histology. Thus, it has valuable diagnostic and prognostic potential for early non-invasive evaluation of radiotherapy.

## Background

Positron Emission Tomography (PET), which is a functional imaging technique, can differentiate between cancer and normal tissues due to differences in the phenotype. The most common approach is based on abnormal metabolic patterns such as glucose metabolism [1]. Fluorine-18-fluorodeoxyglucose (^18^F-FDG) provides a measure of enhanced glucose metabolism by entering the normal glycolytic pathway, but it is accumulated since the 2′-hydroxyl group halts complete catabolism. Therefore, the tracer accumulates at sites of enhanced glycolytic activity. This also means that ^18^F-FDG has limited efficacy for visualizing low-risk prostate cancer due to its low glycolytic activity [2]. However, ^18^F-FDG may be useful in patients with hormone-resistant poorly differentiated cell types [3], since androgen has a modulatory effect on the glucose metabolism of androgen-dependent tumours [4]. Therefore, alternative radiotracers are currently being investigated such as ^18^Fluorine-Fluoromisonidazole (^18^F-FMISO) which is sensitive to hypoxia [5] and ^18^F-Fluorocholine (^18^F-FCH) whose uptake and metabolism are growth related [6]. Both ^18^F-FMISO and ^18^F-FCH represent phenotype traits recognised as hallmarks of malignancy [7, 8]. ^18^F-FMISO is a nitromidazole, an electron acceptor that under hypoxic conditions will be reduced to an alkylating agent and retained in cells inversely related to O_2_ concentration [9]. It was chosen for its promising capabilities for predicting radiotherapy response [6]. ^18^F-FCH is synthesized into the cell membrane in the form of phosphatidylcholine, the primary phospholipid of cell membranes and is a potential marker of cell division [3, 6]. Choline uptake is known to be increased in lung, colorectal, and prostate cancer due to elevated choline kinase activity in these tissues [7]. Under aerobic conditions, androgen-sensitive tumours have shown higher uptake of choline than for ^18^F-FDG [4]. Furthermore, ^11^C-Choline PET in combination to Diffusion-Weighted Magnetic Resonance Imaging (DW-MRI) has significant correlations with the Gleason Score used to classify the malignance of prostatic cancers [10]. Nevertheless, the clinical value of ^18^F-FCH is still controversial regarding the detection of a malignant focus in the prostate as well as local tumour extent (T-staging) [3].

The relatively short half-life of these radiotracers requires on-site or near-site production that is not always available. However, despite accessibility challenges, PET and specific PET-tracers in clinical use have the advantage of better sensitivity above other methods used routinely to detect cancer and cancer metastasis [11]. MRI and Ultrasound (US) provide better spatial resolution than PET, and are more widely available, cheaper, simpler to use and utilizes non-ionizing radiation. In addition to morphology, functional information is achieved using a dynamic acquisition with suitable contrast agents, i.e., dynamic contrast-enhanced MRI (DCE-MRI) and US (DCE-US).

Ideally, a multimodal approach is preferable, but the scant availability of integrated systems, makes such implementation difficult [12]. Therefore, a detailed investigation and comparison of the methods under the same conditions is mandatory to define suitable and reliable imaging biomarkers. The present study aims to assess the effect of radiation treatment on multimodal functional parameters using ^18^F-FMISO PET/CT and ^18^F-FCH PET/CT as biomarkers for hypoxia and proliferation, respectively, as well as DCE-US. Furthermore, to determine in a combined approach the relationship between metabolic parameters and microvascular parameters characterizing the tumour microenvironment. The results of PET/CT and DCE-US were validated by comparing to immunohistochemical parameters such as microvessel density and necrosis.

## Methods

### Animals

This study was approved by the Norwegian Animal Research Authority (Oslo, Norway) and is in agreement with the European Convention for the Protection of Vertebrate Animals used for experiments and other scientific purposes (ETS 123). Experiments were carried out in accordance with the approved protocols.

Healthy male, sexually mature Hsd:Athymic Nude-Foxn1^nu^ mice (6 weeks old, 21–28 g), purchased at Harlan Laboratories (The Netherlands), were used in this study. The mice were hosted under specific pathogen-free conditions at constant temperature and humidity, before and during the experiment. Sterilized food and water were provided ad libitum.

Approximately (2 × 2 × 2) mm^3^ tissue from the CWR22 human androgen-sensitive xenograft was subcutaneously implanted according to procedures previously reported [13]. Tumours were implanted bilaterally so each mouse could be used as control and treated simultaneously. The mice were included in the experiment when their shortest tumour diameter reached 8 mm (the same calliper was used which provided a minimum measurement of 0.1 mm). Nevertheless, not all the mice developed both tumours, and not both tumours reached a suitable size to be included in the experiment. The available tumours were randomly distributed by simple randomization according to the tracer to be used in PET (n_18F-FMISO_ = 14 + n_18F-FCH_ = 14), as well as into control and treated mice. Treated and untreated control mice were euthanized at day 3. Subsequently, the tumour tissue was excised. The distribution of mice per day and modality are specified in Table 1.

**Table 1:** Distribution of mice per day and modality

	^18^F-FMISO GROUP			^18^F-FCH GROUP		
	DCE-US	PET/CT	Histology	DCE-US	PET/CT	Histology
Day 0	n = 13 6 (C)/7 (T)	n = 13 6 (C)/7 (T)	–	n = 14 8 (C)/6 (T)	n = 9 4 (C)/5 (T)	–
Day 3	n = 14 7 (C)/7 (T)	n = 13 6 (C)/7 (T)	n = 13 6 (C)/7 (T)	n = 14 9 (C)/5 (T)	n = 9 4 (C)/5 (T)	n = 14 8 (C)/6 (T)

*n* refers to the number of animals used, while C and T correspond to the control and the treated group, respectively

*C* control, *T* treated

### Radiation treatment

At day 1, the animals were anesthetized and transported to the Department of Oncology and Medical Physics at Haukeland University Hospital for radiation treatment. Injectable anaesthesia was administered subcutaneously using a dose of 0.1 ml/body weight (BW) of a mixture of ketamine hydrochloride (75 mg/kg Ketalar®, Pfizer, Norway), medetomidine hydrochloride (0.1 mg/kg Domitor® vet, Orion Pharma AS, Norway), and saline.

All treated mice were positioned within the radiation field minimizing the exposure to vital organs and control tumours. Subsequently, a Varian Clinac 600C linear accelerator (6 MV x-rays) was used to deliver a single dose of 10 Gy. During the irradiation, the control mice were located outside the treatment vault at approximately 5 m away from the source, except when bilateral tumours were irradiated. In such cases the non-irradiated tumours were positioned as far as possible of the irradiation field.

### Contrast agent and radioactive tracers

^18^F (half-life 109.8 min) was locally produced using PETrace 6 (GE, Lund, Sweden). ^18^F-FMISO and ^18^F-FCH were produced using GE Tracerlab MX with reagent kits from ABX (ABX GmbH, Radeberg, Germany).

Micromarker (Fujifilm Visualsonics, Toronto, Canada) microbubbles were used as contrast agent for DCE-US whose mean diameter ranges from 2.3–2.9 µm [14].

### Imaging acquisition and quantification

PET/CT scans were performed using the integrated PET-CT scanner nanoScan PC PET∙CT (Mediso Medical Imaging Systems Ltd, Budapest, Hungary). The PET field of view (FOV) was 9.5 × 8 cm in axial and transaxial directions respectively, allowing whole-body imaging of mice. The PET detectors consist of LYSO crystals, and acquisition was performed in 1:5 coincidence and normal count mode. PET images were reconstructed using the supplier’s reconstruction algorithm Tera-Tomo 3D (OSEM), with corrections for depth-of-interaction (DOI), radionuclide decay, randoms, crystal dead time, detector normalization, and attenuation correction, and with a detector coincidence mode of 1:3, 4 iterations and 6 subsets, and no filtering. CT images were reconstructed using the RamLak filter. The PET and CT images were co-registered automatically. Images were reconstructed with a voxel size of 0.25 × 0.25 × 0.25 mm^3^ for CT, and 0.4 × 0.4 × 0.4 mm^3^ for PET. PET image data analyses were performed using InterView Fusion version 2.02.055.2010 (Mediso Ldt., Budapest, Hungary).

^18^F-FMISO and ^18^F-FCH PET/CT were acquired pre- (day 0) and post-treatment (day 3). The mice were anesthetized with sevoflurane (Sevoflo, Abbott, Illinois, USA), in 50 % O_2_ and 50 % air (induction 4 %, maintenance 1.5–2 %). The tracers were injected through the tail vein using a 30G (12 mm) insulin needle connected to a 26G (20 mm) catheter tube (BD Vasculon Plus IV, Medinor). Respiratory rate and body temperature were monitored continuously during the image acquisitions and kept at a constant level.

A 120 min interval between tracer injection and the start of the scan was used for ^18^F-FMISO. The DCE-US acquisition was performed immediately before the PET/CT acquisition, following the protocols previously described [15]. In the ^18^F-FCH group, a 30 min interval was used between injection and start of the PET/CT scan. In all cases, the length of the PET/CT scan was 30 min. The SUV were calculated for each tumour as well as on muscle tissue, where SUV = C_PET_(T)/(ID/BW), being C_PET_(T) the measured activity in tissue, ID the injected dose measured in kBq, and BW the mouse body weight in kg. The whole tumour was included in the region of interest (ROI) to allow further analysis of tumour heterogeneity and variance in uptake.

DCE-US quantification was performed by in-house developed software using Matlab R2011b (MathWorks, Inc, Natick, MA, USA) as previously described [15]. Shortly, a region of interest (ROI) was delineated around the entire tumour using the ultrasound images excluding surrounding skin, connective and necrotic tissue. Signal intensities were converted into relative signal intensity (RSI) and fitted to the Brix model [16] using the Levenberg–Marquardt least-squares minimization. Voxelwise estimates of the microvascular parameters *k*_*ep*_, *k*_*el*_, and *A* were provided. Furthermore, the median curve associated to the ROI was also fitted to the Brix model providing an additional estimation of the microvascular parameters per tumour.

In this model, *k*_*ep*_ is the exchange rate constant from the extracellular extravascular space (EES) to plasma; *k*_*el*_ is the eliminating rate constant of the contrast agent from the plasma; *A* is the amplitude of the contrast uptake. *Ak*_*ep*_ obtained as the product of *A* and *k*_*ep*_, reflects permeability surface area per unit volume of vasculature [17].

No constraints were applied to the parameters in the fitting process but no negative values were allowed. Unfitted voxels were set to zero and included in the calculations.

### Immunohistochemistry

Treated and untreated control tumours were taken for immunohistochemistry on day 3. The immunohistochemical preparation of the samples and subsequent analysis of the specimens were performed following the protocols described in detail previously [15]. The tumour vascularity was quantified by both in-house software developed in Matlab 2014b and manually by a blinded investigator. For the manual quantification, microvessel count (MVD) of CD-31 positive structures per 0.5 mm^2^ was assessed in six randomly selected regions. The percentage of necrosis per sample was determined using ImageJ 1.48v (NIH, Bethesda, MD, USA) assessed as the ratio of the number of non-stained DAPI (4′,6-diamidino-2-phenylindole) regions with low autofluorescence signal to the total number of pixels per sampling area.

### Statistical analysis

Statistical analysis was performed in R [18] using in-house developed software. PET parameters, including SUVmean (the average value of the SUV in the whole tumour ROI) and SUV_max_ (single voxel value) in the tumour and muscle, tumour-to-muscle ratio, homogeneity, entropy and intensity variation, were grouped by imaging day: day 0 and day 3, as well as by group: treated and untreated control. Histograms for the microvascular parameters derived from DCE-US were grouped similarly to PET. Mean, median, first and third quartile, as well as skewness and kurtosis were used for the statistical analysis. Similarly, the median curve analysis and the derived median values of the microvascular parameters were tested. The Wilcoxon’s matched-pairs test was used to determine intergroup differences at day 0 and day 3 and to assess intragroup differences from day 0 to day 3. If significant, the provided approximation to the Z value is reported. Kendall’s Tau-b test was employed to assess correlation between parameters derived from PET and DCE-US for each experimental group (^18^F-FMISO and ^18^F-FCH), as well as correlation to histological parameters (MVD and necrosis) derived from the manual and the automatic quantification. The correlation analysis employed the mean value of each parameter per tumour according to Table 1. The Bonferroni adjustment method was employed as correction for multiple testing. A significance level of 5 % was considered in all tests.

## Results

### Volume changes

Tumour volumes were estimated by the same experienced investigator according to calliper measurements as well as DCE-US and PET/CT images. No significant intragroup changes were found longitudinally for any of the experimental groups (^18^F-FMISO and ^18^F-FCH). Similarly, no significant intergroup differences for tumour volumes were detected between treated and control mice at any imaging day.

### Longitudinal changes

#### ^18^F-FMISO PET/CT

**Fig. 1:**
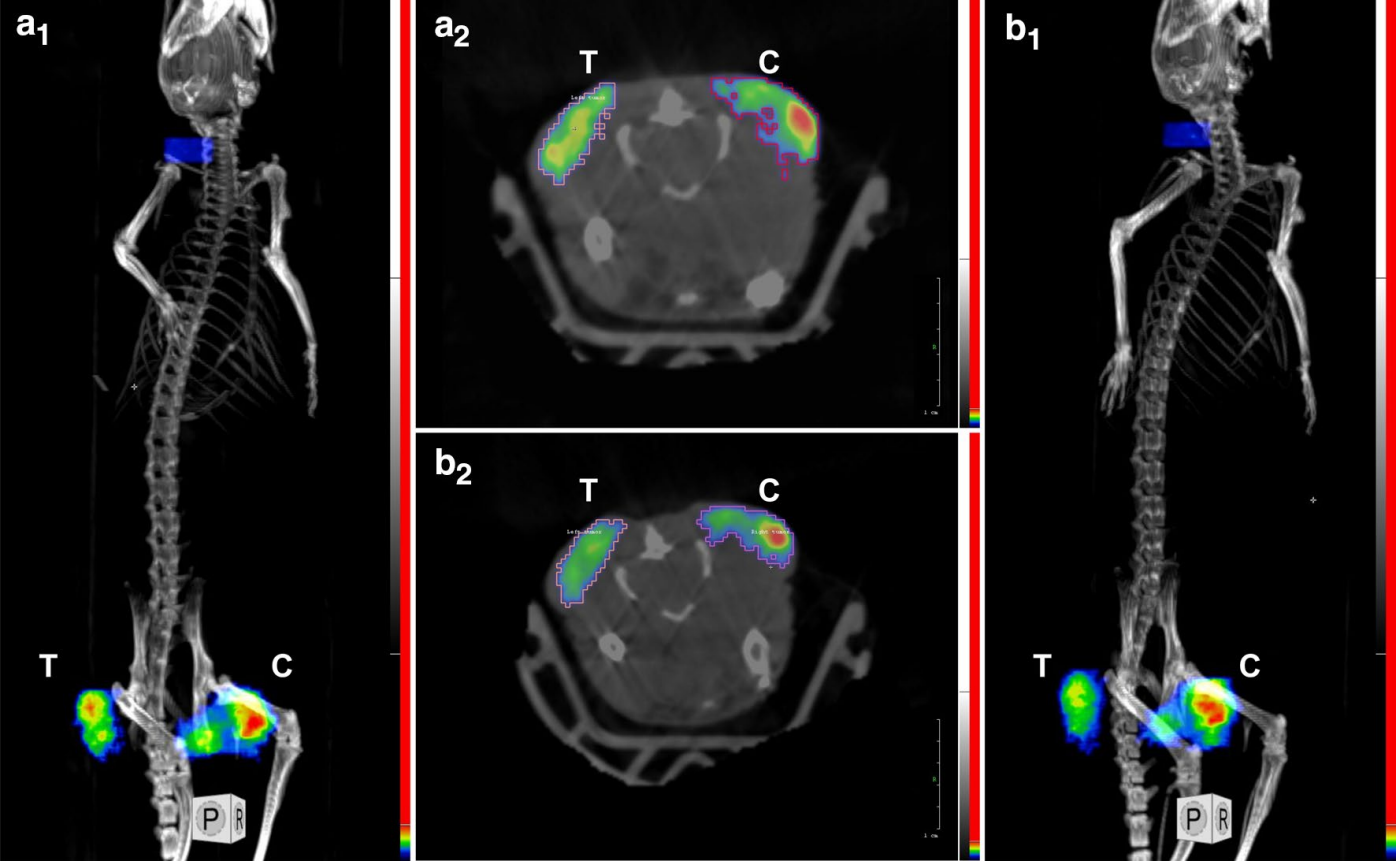
Maximum intensity projections (MIP) for a mouse with a bilateral tumour imaged using ^18^F-FMISO at day 0 (**a1**, **a2**) and day 3 (**b1**, **b2**). The treated (T) and untreated control (C) tumour have been indicated in each image. The *colour* scale goes from *black* (no tracer uptake) to *red* (maximum tracer uptake). The *green colour* indicates intermediate tracer uptake. Scale window is similar in both conditions

Figure 1 displays the maximum intensity projection (MIP) for a mouse imaged using ^18^F-FMISO. At 2 h post-injection, ^18^F-FMISO particularly accumulates in the centre of the tumour due to a hypoxic tumour core. When including all the tumours in the experiment at day 0, significantly decreased SUVmax (Z = 2.7, p < 0.01) and SUVmean (Z = 1.9, p = 0.05) were detected from day 0 to day 3 in the treated group. The SUVmax declined from 1.68 ± 0.16 to 1.33 ± 0.15, while the SUVmean decreased from 0.78 ± 0.12 to 0.63 ± 0.08. When considering treated and control mice separately at day 0, no significant intergroup differences were detected for any parameter. However, decreased SUVmax were found for the treated group but did not reach statistical significance (Z = 1.6, p = 0.1), declining from 1.67 ± 0.15 to 1.33 ± 0.15.

#### ^18^F-FCH PET/CT

**Fig. 2:**
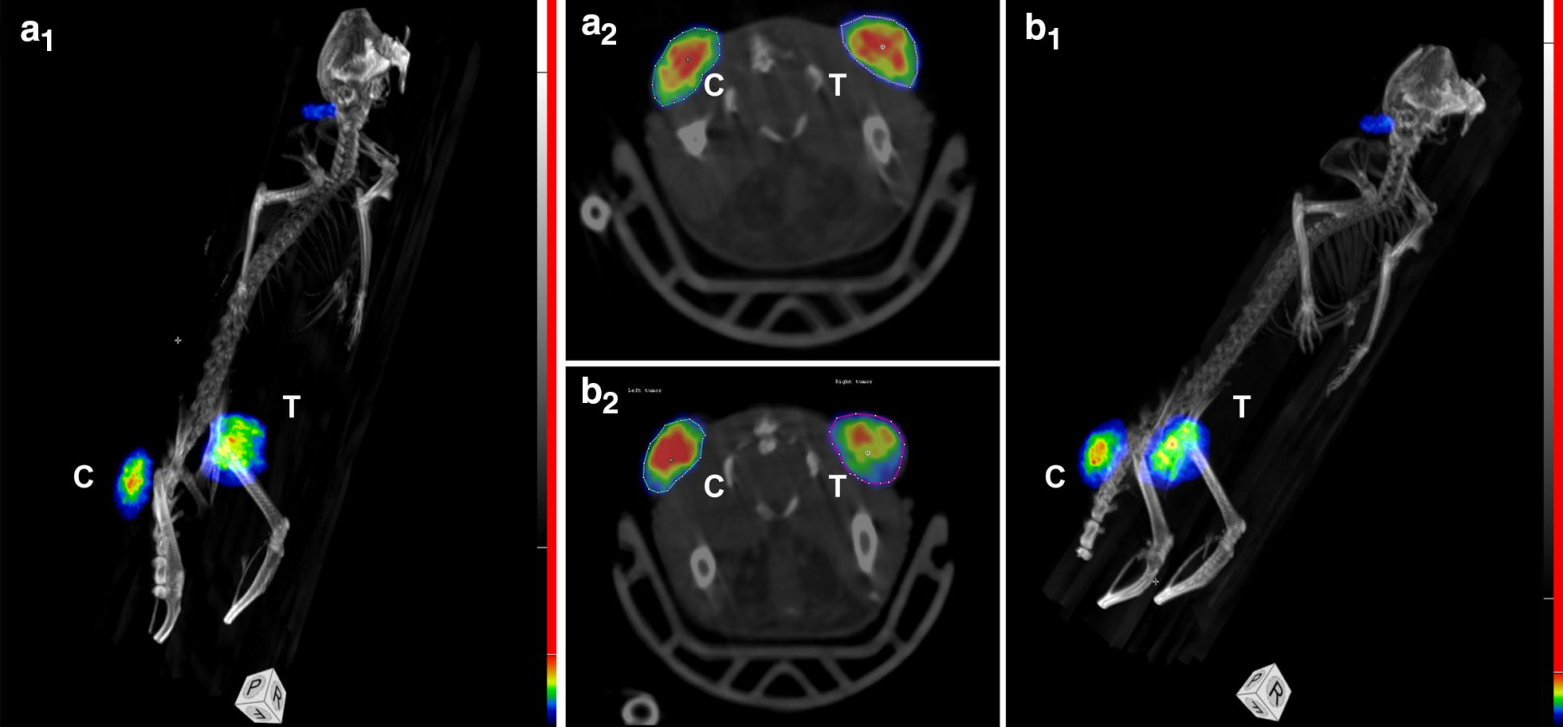
Maximum intensity projections (MIP) for a mouse with a bilateral tumour imaged using ^18^F-FCH at day 0 (**a1**, **a2**) and day 3 (**b1**, **b2**). The treated (T) and untreated control (c) tumour have been indicated in each image. The *colour* scale goes from *black* (no tracer uptake) to *red* (maximum tracer uptake). The *green colour* indicates intermediate tracer uptake. Scale window is similar in both conditions

Figure 2 displays the MIP for a mouse imaged using ^18^F-FCH. In carcinoma tissue, there was a heterogeneous uptake of radiolabelled choline, which reflects the heterogeneous tumour texture of this model. After treatment, no significant changes were found in the SUVmean associated to the tumour or the tumour-to-muscle ratio in any group (treated and control).

#### DCE-US

**Fig. 3:**
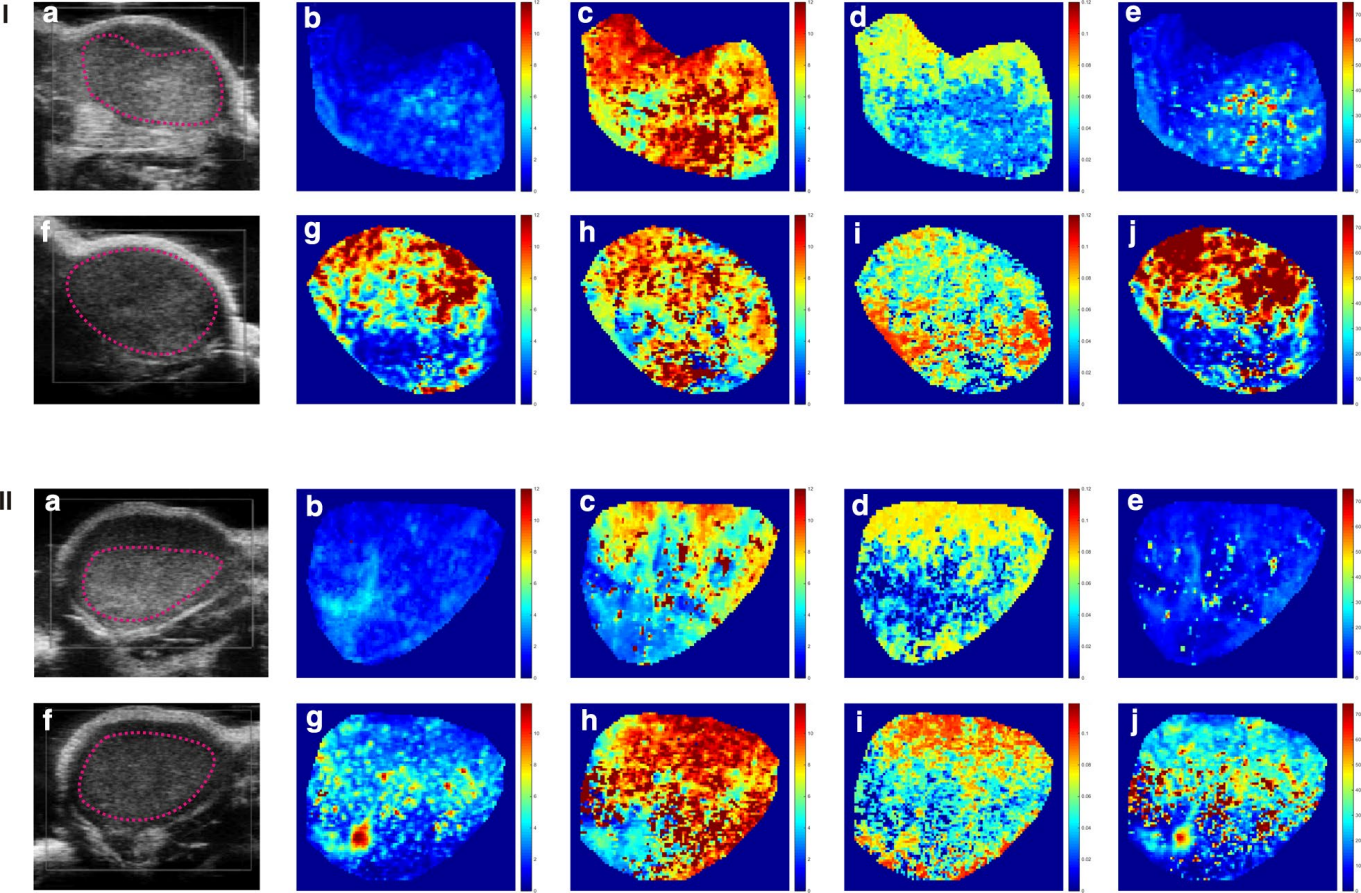
DCE-US analysis of a mouse on the ^18^F-FMISO group showing the control (*panel I*) and the treated (*panel* *II*) tumours at day 0 (*upper row*) and at day 3 (*lower row*). B-mode images (**a**, **f**) as well as the derived parametric maps are displayed: *A* (**b**, **g**), *k*_*ep*_ (**c**, **h**), *k*_*el*_ (**d**, **i**), *Ak*_*ep*_ (**e**, **j**), respectively

The parametric maps derived from DCE-US for a control and a treated tumour on the same mouse are displayed in Fig. 3 at day 0 and day 3, respectively. According to the means, medians, first and third quartiles, no significant longitudinal changes were detected in the microvascular parameters for any of the experimental groups.

Significant differences were observed between treated and control mice at day 3. In the ^18^F-FCH experimental group, the kurtosis of *A* (Z = 2, p < 0.05) was significantly decreased in the control group as compared to the treated one. In the ^18^F-FMISO group, differences did not reach statistical significance although a clear trend was observed. The kurtosis of *Ak*_*ep*_ (Z = 1.7, p = 0.09) was increased for the treated group as compared to the control group, while the third quartile of the *A* (Z = 1.7, p = 0.09) was declined for the treated group.

The analysis of the median curves revealed longitudinal changes in *k*_*el*_ for the treated mice in the ^18^F-FMISO group (Z = 1.9, p < 0.05).

### Correlation to MVD and necrosis

#### ^18^F-FMISO PET/CT

**Fig. 4:**
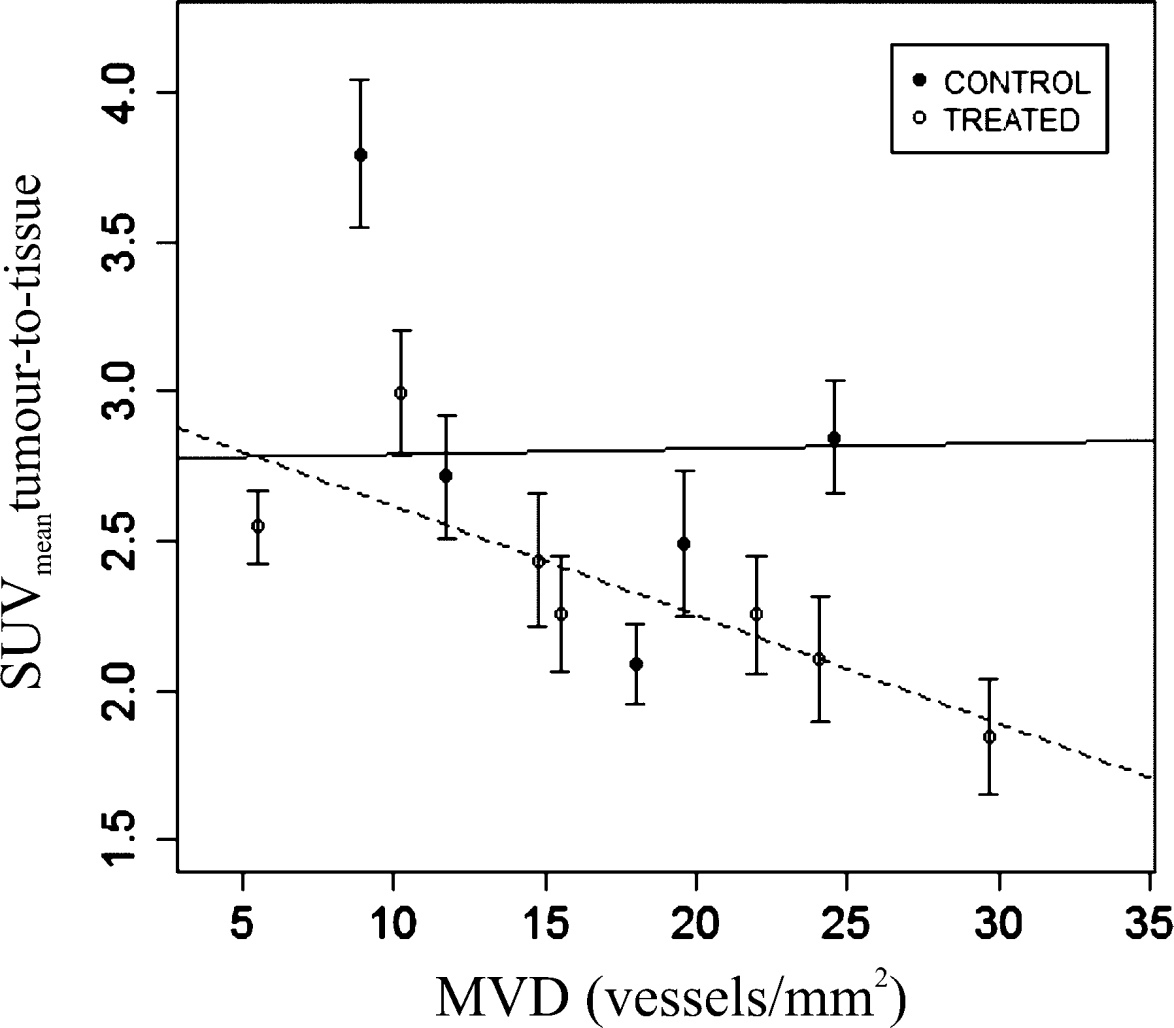
Relationship between the SUVmean of the tumour-to-tissue ratio linked to ^18^F- FMISO PET/CT and the microvessel density for the control (*bold line*) and treated (*dashed line*) groups at day 3. Each data point corresponds to the mean ± standard deviation for each tumour and the correlation is based on these mean values from each experiment

A strong negative correlation was found for the treated group between MVD assessed automatically and the SUVmean of the tumour-to-muscle ratio for the treated group (r_τ_ = −0.9, p < 0.05) as displayed in Fig. 4. The analysis of the median curves showed a strong negative correlation between the MVD assessed manually and the *k*_*el*_ parameter for the control group at day 3 (r_τ_ = −0.9, p < 0.05).

**Fig. 5:**
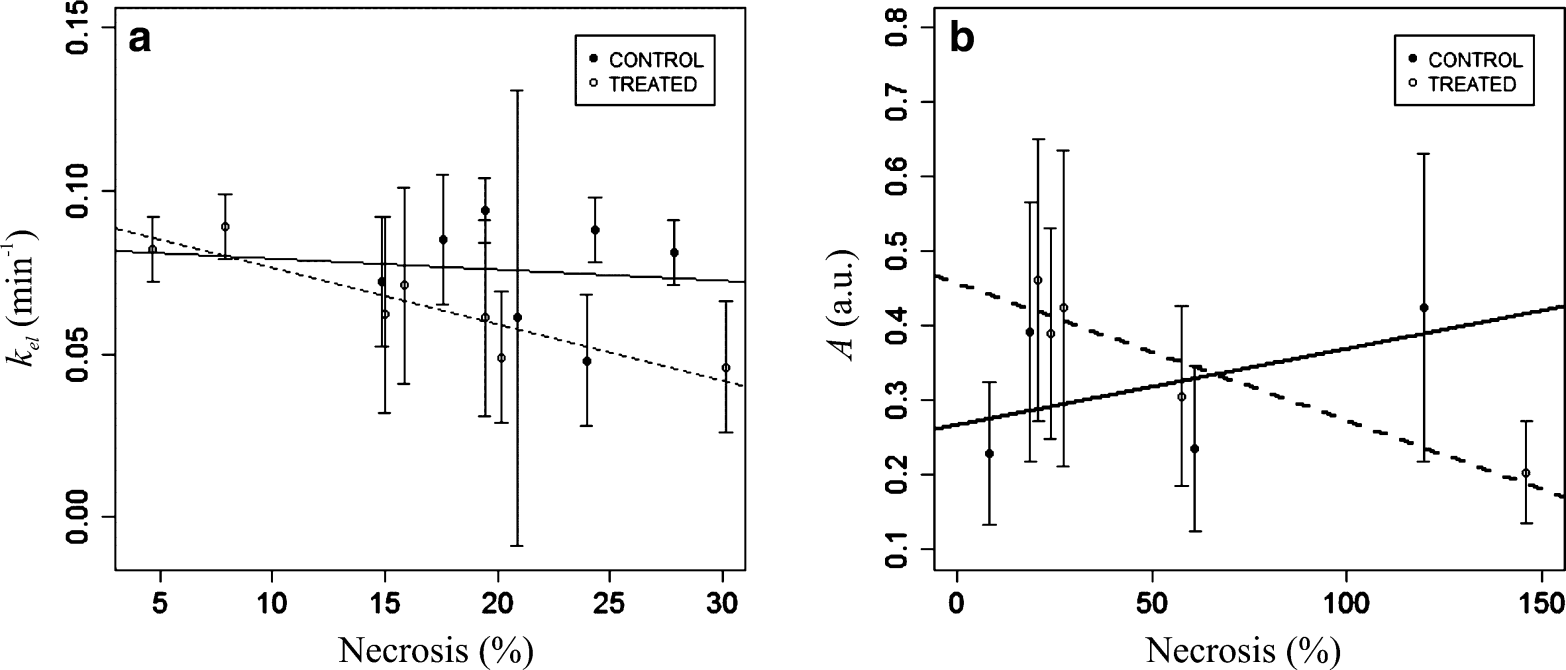
Relationship observed in the ^18^F-FMISO PET/CT group at day 3 between necrosis and **a** the mean values of *k*_*el*_ derived from DCE-US as well as to **b** the mean values of *A* for the control (*bold line*) and treated (*dashed line*) mice. Each data point corresponds to the mean ± standard deviation for each tumour and the correlation is based on these mean values from each experiment

Manual quantification of necrosis correlated strongly to the first quartile of *k*_*el*_ (r_τ_ = −0.8, p < 0.05) for the treated group as can be observed in Fig. 5a. In addition, a correlation was found between the automatic quantification of necrosis and the median values of *A* for the treated group (r_τ_ = −0.8, p < 0.05) as can be seen in Fig. 5b. Manual quantification of necrosis was correlated to the SUVmean of the tumour-to-muscle ratio for ^18^F-FMISO PET/CT, although it was not statistically significant (r_τ_ = 0.8, p = 0.07).

#### ^18^F-FCH PET/CT

No correlation to MVD or necrosis was found for the microvascular parameters derived from DCE-US according to the voxelwise and median analysis. Similarly, no correlation was detected for the functional parameters derived from ^18^F-FCH PET/CT.

In order to discard changes related to differences in tumour size, intra- and intergroup variations in microvessel density and necrosis were assessed. No significant intergroup differences were detected in the histological parameters as evaluated manually or automatically. Similarly, no significant differences were detected between treated and control groups.

### Correlation between PET/CT and DCE-US

#### ^18^F-FMISO PET/CT

**Fig. 6:**
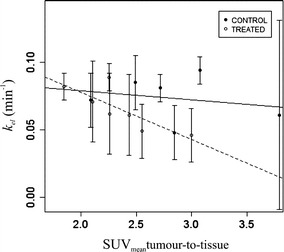
Correlation observed at day 3 between the SUVmean of the tumour-to-tissue ratio linked to ^18^F-FMISO PET/CT and *k*_*el*_ for the control (*bold line*) and treated (*dashed line*) groups. Each data point corresponds to the mean ± standard deviation for each tumour and the correlation is based on these mean values from each experiment

At day 0, the kurtosis of *A* was associated to the SUVmean in the muscle tissue for the control group (r_τ_ = −1, p < 0.05). At day 3, the first quartile of *k*_*el*_ was negatively correlated to the SUVmean of the tumour-to-muscle ratio for the treated group (r_τ_ = −0.8, p < 0.05) as can be seen in Fig. 6. A negative correlation was observed between the first quartile of *A* and the intensity variation derived from PET for the control group, although it was not statistically significant (r_τ_ = −0.9, p = 0.09).

#### ^18^F-FCH PET/CT

At day 0, no correlation was found between the SUVmean of the tumour-to-muscle ratio for ^18^F-FCH and the microvascular parameters evaluated by DCE-US.

At day 3, a negative correlation was found between the homogeneity of the SUVmean for ^18^F-FCH PET and the first quartile of *k*_*ep*_ derived from DCE-US in the treated group, without reaching statistical significance (r_τ_ = −1, p = 0.09).

## Discussion

In the present study functional multimodal imaging was used to assess early response to radiation treatment in a prostate tumour xenograft. Longitudinal changes were only found for ^18^F-FMISO PET/CT, which showed lower maximum uptake (SUVmax) and SUVmean in irradiated tumours, suggesting a decrease in hypoxia. The SUVmax has been shown to predict cancer prognosis [6]. Local recurrence and distant metastasis frequently occur after radiation therapy and these are caused, at least in part, by hypoxia [19]. Diminished blood perfusion and hypoxia in tumours results from abnormal structure [20]. The distribution of oxygen from tumour blood vessels to hypoxic tumour cells is dramatically improved after radiotherapy as a result of the death of well-oxygenated tumour cells and a subsequent decrease in oxygen consumption in cells close to vessels [19]. ^18^F-FMISO is linked to hypoxia which confers resistance to radiation through the hypoxia-inducible factor 1 (HIF-1), and therefore correlates with a poor prognosis [1, 20]. The same trend towards lower ^18^F-FMISO uptake has been reported in bevacizumab treated tumours of the human colon cancer cell line LS174T [5]. Actually, bevacizumab increased hypoxia in the tumour but this could not be detected by ^18^F-FMISO PET or pimonidazole staining. It was concluded that the distribution of the tracer was hampered by the effects of bevacizumab on vascular permeability and perfusion. However, tumours treated with Avastin (bevacizumab) had increased uptake of ^18^F-FMISO and reduced blood flow and vessel permeability [21].

For ^18^F-FCH PET, larger changes were expected in the tumour tissue as cell death of viable, normoxic cells caused by radiotherapy reduce the uptake of ^18^F-FCH, which is related to cell growth. ^18^F-FCH primary sites of uptake are kidney and liver, and normal distribution of ^18^F-FCH is in the salivary glands, liver, spleen, and pancreas; with variable activity in the bowel, in addition to the uptake in the kidneys and bladder from excretion [3]. In this study, heterogeneous uptake of labelled choline in carcinoma tissue has been detected, which depends on choline kinase overexpressed in tumour subregions [22]. A reduced tracer uptake of ^18^F-FCH has been reported in hypoxic conditions [4], and hypoxia was more profound in pre-treatment conditions as demonstrated in this study using the hypoxia tracer. Hypoxic tumours may exhibit low uptake of choline due to poor perfusion as previously suggested or that tumour hypoxia may notably lower choline metabolic trapping in cancer cells. Thus choline tracer retention may be related to tumour oxygenation status though effects on choline kinase activity [7]. Furthermore, a decrease in choline uptake was reported in response to hypoxia, which was markedly declined upon androgen depletion in androgen-sensitive prostate cancer cells [1]. Similarly, a small decrease in ^11^C-choline uptake was reported for the CWR22 tumour model after castration [23]. The reduced uptake of radiolabelled choline and choline derivatives in small-rodent prostate tumour models was attributed to an excess of the nonlabelled precursor dimethylaminoethanol [23].

In this study significant correlations between techniques as well as to histological parameters have been detected. For the ^18^F-FMISO group, the SUVmean of the tumour-to-muscle ratio was negatively correlated to MVD for the treated group, while the correlation to necrosis was not statistically significant. Furthermore, the microvascular parameter *k*_*el*_ was found negatively correlated to the SUVmean of the tumour-to-muscle ratio, necrosis, and MVD. Tumour heterogeneity has been included in the study by using the descriptive analysis of the histograms associated to PET/CT and DCE-US. Imbalance between oxygen supply and consumption in a malignant solid tumour is a major cause of heterogeneity [19]. Kurtosis, skewness, percentiles and their changes have been shown to be quantitative markers of tumour heterogeneity with a direct correlation to the underlying structural, physiological, molecular and metabolic changes [24]. The kurtosis, homogeneity and intensity variation derived from PET/CT, were related to the microvascular parameters derived from DCE-US. Particularly, correlations to the microvascular parameter *A* and kurtosis were observed pre-treatment or for the control group, whereas correlation to intensity variation was related to the treated group. A lesser degree of spatial heterogeneity in hypoxia may be associated to a more localized and well-demarcated process, for instance the gradient in vascularization, which decreases towards the centre of the tumour. In contrast a diffuse hypoxic process indicates a response to hypoxic stress with associated biological changes [25].

The reduced number of mice in the ^18^F-FCH PET/CT group may have prevented the observation of additional correlations between metabolic and microvascular parameters. Nevertheless, a strong relationship to kurtosis was found for the amplitude of the contrast uptake *A* that was not related to the treatment. The treated group exhibit a negative relationship between homogeneity and *k*_*ep*_ that did not reach statistical significance (data not shown). Kinetic analysis of dynamic ^18^F-FDG using the same tumour model, reported heterogeneous intragroup parameters variation 24 h after radiation treatment with 7.5 Gy in contrast to the homogeneous distribution presented in the control group [26]. In addition, radiation treatment increases permeability facilitating the diffusion of injected substances into the EES [27]. Clinically, the value of ^18^F-FCH PET in the stage of prostate cancer is controversial and highly variable [3]. The common sextant biopsy method is prone to sampling errors, however, prostate sextants with malignant involvement demonstrated stable or increasing ^18^F-FCH uptake over time while most benign tissue demonstrated a corresponding decrease [28]. These observations are in correspondence with our results. In addition, correlation between SUVmax and sextants with maximal tumour infiltration has been reported, as well as a significant difference in SUVmax between malignant and benign regions [28, 29].

Earlier, 24 h after treatment with a single dose of 7.5 Gy, we detected longitudinal changes in the microvascular parameter *k*_*el*_ derived from DCE-US as assessed by the median values [15]. In addition, MVD and necrosis were correlated to *k*_*ep*_ for the treated group only in high intensity areas of enhancement. In the present study, 48 h after a dose of 10 Gy, we identified longitudinal changes for *k*_*el*_ only in the analysis of the median curves and exclusively for the ^18^F-FMISO group. In addition, necrosis was negatively correlated to *A* and *k*_*el*_ for the treated group, whereas *k*_*el*_ was linked to MVD for the control group. Thus, *k*_*el*_ could indicate the tumour blood supply and vascular integrity. We associate the differences observed, in comparison to the earlier study, to the increased time post-treatment and the increased radiation dose. These two factors affect the window of normalization which occurs after treatment regarding the destruction of vessels. Intratumour HIF-1 activity has been shown to reach a minimum level at an early phase and subsequently increase to a plateau in a late phase. The timing and duration of the activation was shown to be dependent on the dose of radiation [19]. Radiation treatment has a better outcome when given during the normalization window in which the perfusion is improved and the hypoxia reduced due to direct damage or killing of well-oxygenated tumour endothelial cells and the subsequent decrease in oxygen consumption (tumour reoxygenation) [19, 20, 30, 31].

However, a different normalization window does not explain the lack of correlation between the histopathological findings and the microvascular parameters generated from DCE-US for the ^18^F-FCH group. The estimation of the microvascular parameters *k*_*ep*_ and *k*_*el*_ derived from DCE-US in the ^18^F-FMISO and the ^18^F-FCH group differed significantly between the median and the voxelwise analysis pre-treatment. The same effect was noticed post-treatment for the control group but not for the treated one (data not shown). A small sample size may have had an impact on the estimation of the microvascular parameters, and therefore, failed to show clearly the effects of the treatment. However, we cannot discard other factors associated to the experimental conditions.

## Conclusions

The multimodal approach ^18^F-FMISO PET/CT and DCE-US seems to provide reliable, non-invasive biomarkers in the assessment of both microvasculature and necrosis as validated by histology. Particularly, the SUVmean of the tumour-to-muscle ratio derived from ^18^F-FMISO PET/CT is negatively correlated to MVD and positively linked to necrosis. *k*_*el*_ derived from DCE-US present promising capabilities as a suitable imaging biomarker for radiation treatment monitoring since it is negatively linked to necrosis and MVD. Moreover, this parameter correlates to the SUVmean of the tumour-to-muscle ratio derived from ^18^F-FMISO PET/CT. This is of importance considering that the combination of information from multimodal approaches will improve the sensitivity and specificity of the diagnostic process.
