# Erratum to: Radiation treatment monitoring using multimodal functional imaging: PET/CT (^18^F-Fluoromisonidazole & ^18^F-Fluorocholine) and DCE-US

**DOI:** 10.1186/s12967-016-0813-0

**Published:** 2016-02-24

**Authors:** Natalia Arteaga-Marrero, Cecilie Brekke Rygh, Jose F. Mainou-Gomez, Tom C. H. Adamsen, Nataliya Lutay, Rolf K. Reed, Dag R. Olsen

**Affiliations:** Department of Physics and Technology, University of Bergen, P.O. Box 7803, 5020 Bergen, Norway; Department of Biomedicine, University of Bergen, Bergen, Norway; Department of Health Sciences, Bergen University College, Bergen, Norway; Department of Clinical Medicine, University of Bergen, Bergen, Norway; Department of Radiology, Haukeland University Hospital, Bergen, Norway; Department of Chemistry, University of Bergen, Bergen, Norway; Division of Dermatology and Venereology, Department of Clinical Sciences, Lund University, Lund, Sweden; Centre for Cancer Biomarkers (CCBIO), University of Bergen, Bergen, Norway

## Erratum to: J Transl Med (2015) 13:383 DOI 10.1186/s12967-015-0708-5

It has come to the publisher’s attention that the original version of this article [1] unfortunately contained an incorrect panel for Figure 5b. Figure 1 published in this Erratum should replace Figure 5 of the original article.

**Fig. 1:**
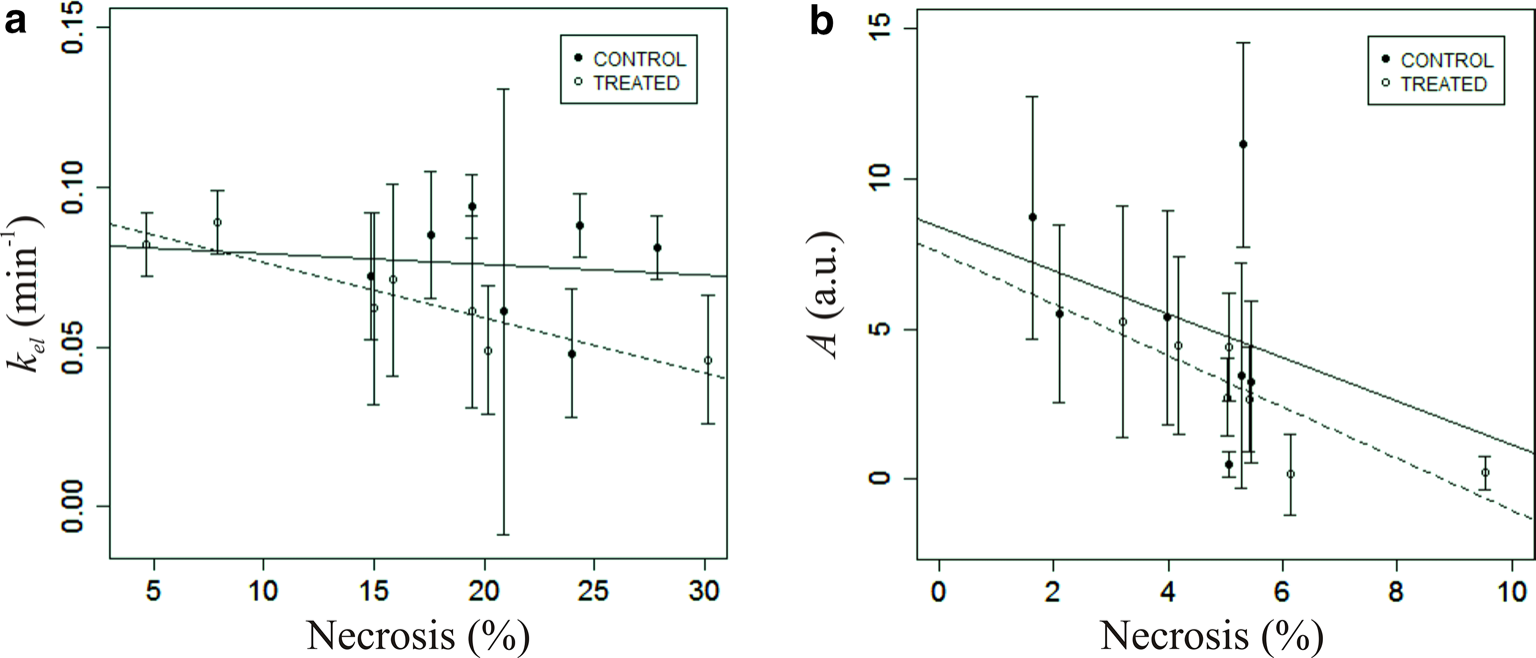
Relationship observed in the ^18^F-FMISO PET/CT group at day 3 between necrosis and **a** the mean values of *k*_*el*_ derived from DCE-US as well as to **b** the mean values of *A* for the control (*bold line*) and treated (*dashed line*) mice. Each data point corresponds to the mean ± standard deviation for each tumour and the correlation is based on these mean values from each experiment
